# Comparative Transcriptome Analysis of Primary Roots of *Brassica napus* Seedlings with Extremely Different Primary Root Lengths Using RNA Sequencing

**DOI:** 10.3389/fpls.2016.01238

**Published:** 2016-08-19

**Authors:** Xiaoling Dun, Zhangsheng Tao, Jie Wang, Xinfa Wang, Guihua Liu, Hanzhong Wang

**Affiliations:** Rapeseed Genetics and Breeding, Oil Crops Research Institute of the Chinese Academy of Agricultural Sciences/Key Laboratory of Biology and Genetic Improvement of Oil Crops, Ministry of AgricultureWuhan, China

**Keywords:** *Brassica napus*, primary root development, RNA-seq, differentially expressed gene, transcription factor, metabolism, hormone, signaling

## Abstract

Primary root (PR) development is a crucial developmental process that is essential for plant survival. The elucidation of the PR transcriptome provides insight into the genetic mechanism controlling PR development in crops. In this study, we performed a comparative transcriptome analysis to investigate the genome-wide gene expression profiles of the seedling PRs of four *Brassica napus* genotypes that were divided into two groups, short group (D43 and D61), and long group (D69 and D72), according to their extremely different primary root lengths (PRLs). The results generated 55,341,366–64,631,336 clean reads aligned to 62,562 genes (61.9% of the current annotated genes) in the *B. napus* genome. We provide evidence that at least 44,986 genes are actively expressed in the *B. napus* PR. The majority of the genes that were expressed during seedling PR development were associated with metabolism, cellular processes, response to stimulus, biological regulation, and signaling. Using a pairwise comparison approach, 509 differentially expressed genes (DEGs; absolute value of log2 fold-change ≥1 and *p* ≤ 0.05) between the long and short groups were revealed, including phytohormone-related genes, protein kinases and phosphatases, oxygenase, cytochrome P450 proteins, etc. Combining GO functional category, KEGG, and MapMan pathway analyses indicated that the DEGs involved in cell wall metabolism, carbohydrate metabolism, lipid metabolism, secondary metabolism, protein modification and degradation, hormone pathways and signaling pathways were the main causes of the observed PRL differences. We also identified 16 differentially expressed transcription factors (TFs) involved in PR development. Taken together, these transcriptomic datasets may serve as a foundation for the identification of candidate genes and may provide valuable information for understanding the molecular and cellular events related to PR development.

## Introduction

Root system architecture (RSA), which is defined as the three-dimensional distribution of a root system within the soil, consists of root morphology, topology, and distribution and determines root plasticity to capture nutrients and water in a constantly changing environment (Lynch, 1995; Rogers and Benfey, 2015). The Brassicaceae species, including the model plant *Arabidopsis thaliana* and the genus *Brassica*, many of which are globally cultivated crops or vegetables (Cheng et al., 2013), have a complex RSA that is mainly composed of the primary root (PR) and the lateral roots (LRs) emerging from the PR (Santosh et al., 2015).

PR development represents a crucial developmental process that is essential for plant survival. In *Arabidopsis*, the morphogenesis of PR originates from the establishment of the embryonic body, which is generated by two main meristems in plants, namely, the shoot apical meristem (SAM) and the root apical meristem (RAM; Lau et al., 2012; Petricka et al., 2012). The PR develops from the division and differentiation of stem cell niche (SCN) cells that reside in the RAM (Giehl et al., 2014). The SCN is formed by the quiescent center (QC) and the surrounding stem cells (De Smet et al., 2015). The QC functions in the maintenance of adjacent meristematic stem cells (Drisch and Stahl, 2015). The stem cells divide through different pathways to generate new stem cells surrounding the QC cells, as well as daughter cells that undergo further cell division to generate vascular, cortex-endodermis, epidermis-lateral root cap, and columella initials from the outside to the inside of the PR (Petricka et al., 2012). When new cells progress further away from the SCN, they stop dividing and undergo differentiation and elongation to form three distinct regions, the meristematic zone (MZ), the elongation zone (EZ), and the differentiation zone (DZ), which in turn determine the basis for the growth, development, and regeneration of the roots (De Smet et al., 2015). The position of the MZ determines the size of the RAM and is thus directly related to the root growth rate (Petricka et al., 2012). In the EZ, cells increase in length and width by cell expansion and elongation; this contributes to root growth (Verbelen et al., 2006). Once the cells in the EZ reach their final size, they form the DZ, which has specialized characteristics and functions, such as water and solute uptake (Petricka et al., 2012). One notable feature of the DZ is the emergence of root hairs (Drisch and Stahl, 2015).

The destiny of stem cells, which determine the length and direction of the PR, involves cell division, differentiation, expansion, and elongation and requires the accumulation of biological substances, such as proteins, carbohydrates, and lipids (Braybrook and Harada, 2008; Park and Harada, 2008). In addition to phytohormones, intercellular signaling molecules (e.g., ion, protein kinases, and phosphatases) and their respective receptors, as well as specific transcription factors (TFs), are essential for the maintenance of stem cell homeostasis in the PR and have been extensively studied in *Arabidopsis* (Drisch and Stahl, 2015). Auxin plays a major role in the regulation of PR development, which largely depends on its concentration gradient along the root axis (Sabatini et al., 1999; Hernández-Barrera et al., 2011). Several best known genes that influence the maintenance and differentiation of RAM have been identified in *Arabidopsis*. For example, the *PIN-FORMED* (*PIN*) genes, encoding the auxin efflux carriers, mediate polar auxin transport and regulate cell division and cell expansion in the PR (Blilou et al., 2005). Auxin activates the AP2-domain transcription factors *PLETHORA1* (*PLT1*) and *PLT2*, which determine root stem cell positioning and differentiation based on their differential expression in the SCN (Aida et al., 2004; Galinha et al., 2007). Similar to the *PLT* genes, the transcription factors *SHORT ROOT* (*SHR*) and *SCARECROW* (*SCR*) are required for the maintenance of the SCN and control cell division in the RAM by directly activating a D-type cyclin D6;1 (*CYCD6;1*; Sozzani et al., 2010; Cruz-Ramírez et al., 2012; Wachsman et al., 2015).

Besides gene functional analysis using mutagenesis, global gene expression profiles offer new opportunities to understand the underlying mechanisms of PR development at the whole-genome level. RNA sequencing (RNA-seq), a high-throughput and deep-sequencing technology, has become a useful tool for the analysis of gene expression patterns and developmental pathways of complex traits in crops (Mortazavi et al., 2008), such as the genetic basis of salt tolerance in rapeseed leaves and roots (Yong et al., 2014), seed development in *Pisum sativum* (Liu et al., 2015), embryo development in *B. rapa* (Zhang et al., 2014), and drought stress and rehydration in tomato (Iovieno et al., 2016). Oilseed rape (*B. napus*), a globally cultivated crop from the *Brassica* genus, is an important vegetable oil source that has been used for human consumption. Understanding the molecular mechanisms that affect PR development is essential for RSA determination, nutrient efficiency, and yield potential for rapeseed. In the present study, we used RNA-seq to investigate the genome-wide gene expression profile of the PR at the seedling stage between two groups of *B. napus* with extreme PR lengths (PRLs). These transcriptome datasets provide a foundation for the expression analysis of candidate genes and valuable information for understanding the molecular and cellular events that are related to PR development.

## Methods

### Plant materials

Forty natural *B. napus* accessions were selected from an association population with natural gerplasm and cultivars (Li et al., 2014) based on their great genetic diversities. Detailed information for these accessions, including their accession number and genetic distance, is provided in Table S1. Plants of the selected *B. napus* accessions were grown in a greenhouse (60–80% relative humidity) under cycles of 16 h of light at 24°C followed by 8 h of dark at 20°C using the hydroponic test. Rapeseed seeds were first sterilized with 10% (v/v) hydrogen peroxide for 5 min. After being soaked with pure water for 8 h, plump seeds were selected, sown on medical gauzes in a beaker for 2 days at 28°C in the dark, and then grown in the light for 4 days. To maintain the moisture and nutrient for seed germination, the beaker was filled with a quarter of modified Hoagland's nutrient solution (pH 5.8), which contained the following macronutrients: 10 mM NO_3_, 1 mM PO_4_, 6 mM K, 5 mM Ca, 2 mM Mg, and 2 mM SO_4_ and the following micronutrients: 50 μM Fe-EDTA, 230 μM H_3_BO_3_, 3.5 μM Zn, 1.85 μM MoO_4_, 1.6 μM Cu, and 0.7 μM Mn. Then, the uniform seedlings were transplanted to half-strength modified Hoagland solution (pH 5.8). The solution was replaced with completely modified Hoagland solution (pH 5.8) once a week.

After growth for 5, 10, and 18 days, 15 plants from each accession (5 plants per replicate) were sampled to determine the PRL. Based on PRL determination, four *B. napus* inbred lines with extremely phenotypic differences in PRL were selected and divided into two groups: D43 and D61 (short group) had a shorter PRL, and D69 and D72 (long group) had a longer PRL. To further confirm the differences between the two groups, the experiment was repeated using the paper roll growth method as described by Abdel-Ghani et al. (2015).

### Sample collection, RNA assessment, and illumina sequencing

To avoid the influence of lateral roots, the PRs of D43, D61, D69, and D72 were collected at 4 days after germination, when the lateral roots had not yet formed. A total of 25 plants for each accession were collected for total RNA extraction. All of the samples in a tube were fully mixed for total RNA extraction using the TRIzol reagent (Invitrogen, USA) and treated with RNase-free DNase I (Thermo Scientific, USA) to remove any contaminating DNA. The quality and integrity of the extracted RNAs were assessed using the NanoDrop 2000 spectrophotometer (Thermo Scientific, USA) and the RNA Nano 6000 Assay Kit of the Bioanalyzer 2100 system. The threshold of the RNA integrity number (RIN) was set to at least 8. After the quality assessment, 3 μg of RNA per sample was further processed by the purification of polyA-containing mRNA, mRNA fragmentation, double-stranded cDNA synthesis, and polymerase chain reaction (PCR) amplification using the Illumina TruSeq RNA Sample Preparation Kit (Illumina, USA) according to the manufacturer's protocol. The final cDNA libraries were sequenced on an Illumina HiSeq™ 2000 platform by the Oebiotech Company in Shanghai, China, and 125 paired-end base pair (bp) reads were generated.

Raw data (raw reads) in fastq format were processed by running a self-written Perl script in NGS QC Toolkit v2.3.3 software. In this process, clean reads were generated by removing adapter sequences, low-quality reads, and uncertain bases (N). At the same time, the descriptive statistics for the clean data, such as Q20, Q30, GC content, and sequence duplication level, were calculated. Then, the clean reads were used in the subsequent analysis. Detailed information for the self-written Perl script is provided in Supplementary Material Presentation 1. The raw data was submitted into database of the National Center for Biotechnology Information (Accession No. SRX1981396).

### Sequence alignment to the *B. napus* reference genome

The *B. napus* reference genome and corresponding gene annotation files were downloaded directly from the *B. napus* genome website (Chalhoub et al., 2014; http://www.genoscope.cns.fr/brassicanapus/data/). The clean reads were mapped to the reference genome using TopHat2. The self-written Perl script for the alignment is provided in Supplementary Material Presentation 1. The Bowtie2 software was used to count the number of reads that were mapped to each gene within the gene model annotation file. To measure the level of gene expression, the FPKM (fragments per kilobase per million reads) value of each gene was calculated based on the length of the gene and the read count mapped to this gene. This method avoids the effects caused by the transcript size and sequencing depth of the four libraries.

### Gene functional analysis

To determine the biological significance of the detected genes, transcripts in all of the samples were searched by BLASTN with an *E* < 10^−5^ against TAIR (http://www.arabidopsis.org/Blast/index.jsp). Then, the unigenes (AGI identifiers) were used to annotate these detected genes. Based on the results of a homology search with *Arabidopsis* genes in the Plant and Transcription Factor Database (PTFB; http://planttfdb.cbi.pku.edu.cn/), the putative TFs in the detected genes were described. A GO enrichment analysis of detected genes was conducted according to molecular function, biological process, and cellular component ontologies (http://www.geneontology.org/) using Blast2GO. KEGG pathways were assigned to the detected genes in the KEGG database (). MapMan software was used to provide a graphical overview of the metabolic and regulatory pathways for the detected genes as described by Gao et al. (2013).

### Identification and analysis of DEGs

The DEGseq method was used to identify DEGs using the DESeq R package. The false discovery rate (FDR) was used to determine the threshold of the *p*-value. The integration of a *p* ≤ 0.05 and an absolute value of log2 ratio ≥1 were used to identify DEGs. First, the expression level of the detected genes was compared in parallel between libraries from the two groups. Then, the common DEGs were obtained from four comparisons. To group DEGs with similar expression patterns, a hierarchical clustering was generated using the normalized FPKM values from each library. GO, KEGG and MapMan analyses were also performed for the functional analysis of DEGs. Furthermore, the REVIGO tool available at http://revigo.irb.hr/ was used to provide more functional relevance for the DEGs by filtering subsets of the GO categories with reduced redundancy under the user-provided cutoff value *C* = 0.4, as described by Supek et al. (2011).

### Real-time reverse transcription PCR

To validate the differential expression pattern of DEGs obtained by Illumina sequencing, qRT-PCR of all of 16 differentially expressed TFs and 21 randomly chosen DEGs was conducted using the total RNA extracted from PRs of six 4-day-old plants of each accession. Gene-specific primers were designed using Primer 3 (http://fokker.wi.mit.edu/primer3/input.htm), and the primer sequences are presented in Table S2. Approximately 3.0 μg of total RNA for each sample was reversed following the instructions of a reverse transcription kit (Cat. M1701, Promega), and first-strand cDNA was amplified according to the instructions for RealMasterMix (SYBR Green [FP202]; TIANGEN). The *B. napus ACTIN2* gene-specific primer (Table S2) was used as a control to normalize the expression data. The results were analyzed using CFX Manager Software using the 2^−ΔΔCT^ method.

## Results and discussion

### Four *B. napus* accessions have dramatically different PRLs at the seedling stage

**Figure 1:**
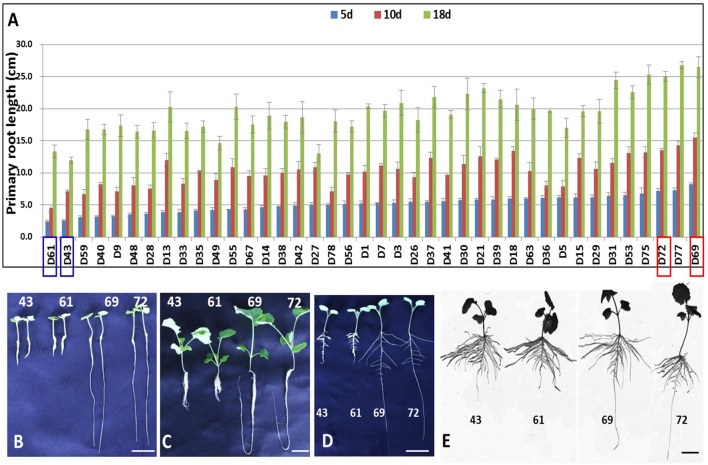
**Primary root length analysis of the samples used in this study**. **(A)** PRL analysis of 40 *B. napus* accessions after growth for 5, 10, and 18 days by a hydroponics test. **(B,C)** The phenotypes of D43, D61, D69, and D72 with extremely different PRLs after growing for 5 days **(B)** and 18 days **(C)** by hydroponics test. **(D,E)** The phenotypes of D43, D61, D69, and D72 after 7 days **(D)** and 15 days **(E)** of growth under a paper roll. Scale bars = 2 cm **(B,D,E)** and 3 cm **(C)**.

To select representative genotypes of *B. napus* for the comprehensive characterization of genes associated with PR development, the PRLs of 40 natural *B. napus* accessions were investigated after growth for 5, 10, and 18 days by a hydroponic test. The phenotypic values for the 40 accessions were detected in three independent replicates. Based on the phenotypic values, four *B. napus* genotypes (D43, D61, D69, and D72) showing extreme PRL values were selected for subsequent analysis. D43 and D61 had a relatively shorter PRL, whereas D69 and D72 had a longer PRL (Figures 1A–C). A two-way ANOVA was performed to test the PRL differences of the four selected accessions in the three stages. The two accessions with longer PRLs differed significantly from the other accessions with shorter PRL at the three stages (*MS* = 27.56, *F* = 88.2, *p* = 0.011; *MS* = 75.69, *F* = 28.12, *p* = 0.034; and *MS* = 122.32, *F* = 19.24, *p* = 0.048, respectively). To accurately evaluate the four genotypes, the physiological changes in PRLs were compared in parallel under paper roll growth conditions. In contrast, the PRLs of genotypes D69 and D72 were relatively higher than those of genotypes D43 and D61 (Figures 1D–E). To facilitate comparison and analysis, the four *B. napus* genotypes were divided into two groups for PRL: D43 and D61 (short group) and D69 and D72 (long group). Based on the marked differences in PRL, we assessed the gene expression of all four genotypes to provide a more comprehensive overview of the PR gene profiles in *B. napus*.

### Illumina sequencing and mapping of the sequence reads

Four cDNA libraries were separately constructed using polyA^+^ RNA, which was isolated from hydroponically grown 4-day-old primary roots of D43, D61, D69, and D72. RNA-seq generated 55,454,800 (D43), 64,631,336 (D61), 55,341,366 (D69), and 58,596,198 (D72) clean reads after trimming and filtering (Table 1). The Phred quality score of >30 (Q30) was >90%, and the guanine-cytosine (GC) content was consistently 47% for the four samples (Table 1), suggesting high-quality sequencing. Approximately 70.67–75.27% of the clean reads were successfully mapped to the *B. napus* genome (http://www.genoscope.cns.fr/colza-ggb/data/) using the TopHat2 software, and 56.45–65.33% of them matched to unique genomic locations (Table 1). The distribution of gene coverage was analyzed in each library using the percentage of a gene covered by unique mapping reads to the total number of bases in that gene. Figure S1 shows that the most abundant category in the four libraries included genes with 90–100% coverage by uniquely mapping reads, and the other nine categories displayed similar percentages of matched genes among the four genotypes. These results suggested that the RNA-Seq data used in the present study was highly reliable.

**Table 1:** **Statistics of RNA-Seq for primary roots of four ***B. napus*** genotypes referring to ***B. napus*** genome**.

**Genotypes**	**D43**	**D61**	**D69**	**D72**
Raw reads	55,939,062	65,046,902	55,709,670	59,033,066
Clean reads	55,454,800	64,631,336	55,341,366	58,596,198
Q30(%)	90.04%	90.98%	90.94%	90.45%
GC content(%)	47.00%	47.00%	47.00%	47.00%
Total mapped	70.67%	72.48%	75.27%	72.78%
Uniquely mapped	63.70%	65.33%	56.45%	64.79%
Multiple mapped	6.97%	7.14%	18.82%	7.99%
Unmapped reads	29.33%	27.52%	24.73%	27.22%
The number of detected genes in libraries	53047	54081	54501	53386

*Numbers in the last column represent the genes that were detected in each library by alignment to the current annotated B. napus genomes*.

In total, 53,047, 54,081, 54,501, and 53,386 genes (ranging from 100 to ≥2000 bp) were expressed in the PRs of D43, D61, D69, and D72, respectively (Table 1). A Venn diagram was constructed to show the genes that were shared between the samples or in each sample. Of these genes, 44,986 genes were expressed in the four samples (Figure S2). Consequently, 62,562 genes accounting for 61.9% (62,562/101,040) of the annotated gene models in the *B. napus* genome were expressed in the PRs across the four libraries (Figure S2).

### Functional annotation of the PR transcriptome in *B. napus*

BLASTN was performed against the *Arabidopsis* Information Resource (TAIR, http://www.arabidopsis.org/Blast/index.jsp) to annotate the detected genes based on the high coding sequence homology (~85%) between *B. napus* and *Arabidopsis* (Chalhoub et al., 2014). Of the 62,562 detected genes, 50,195 (80.23%) hit homologs in *A. thaliana*, with an *E* < 10^−5^ for nucleic acids, and were thus annotated. For the transcript profiling of *B. napus* PRs, the normalized gene expression levels of each detected gene were analyzed based on the fragments per kilobase per million reads (FPKM). The detailed statistical analysis of the detected genes involved in the FPKM value in each library, the homologs, and the annotated information in *A. thaliana* are shown in Table S3. Based on the results of search in the PTFB, a total of 5102 putative TF genes out of 62,562 genes representing 59 TF families were identified in the seedling PR of *B. napus* (Table S4). The majority of these putative TFs belonged to the MYB, bHLH, ERF, NAC, C2H2, WRKY, bZIP, and MIKC families, in which the MYB and bHLH TFs were overrepresented (Table S4).

**Figure 2:**
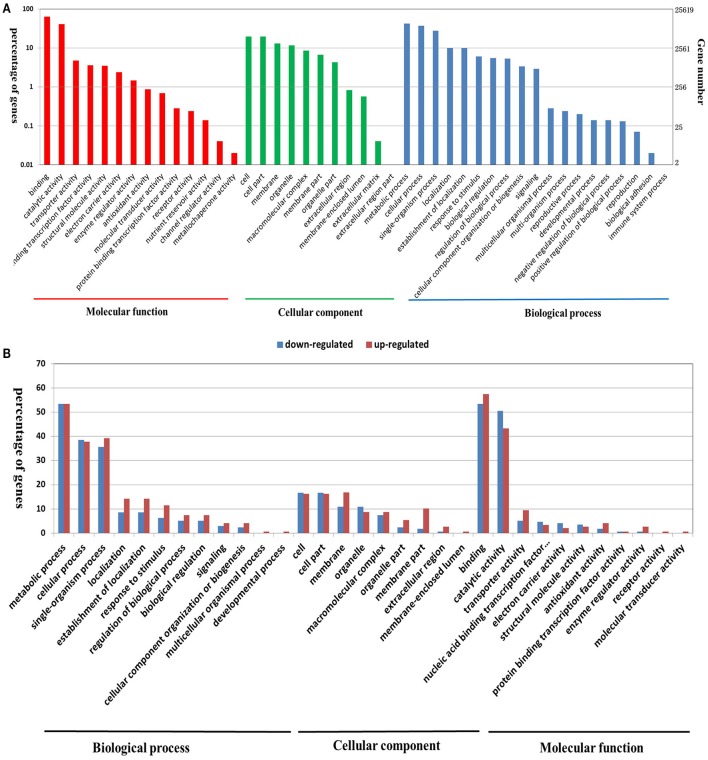
**Functional annotation of all of the detected genes in ***B. napus*** PRs (A), and DEGs between the two PR groups (B) based on GO classification**. The results are summarized under three top-level ontologies: biological process, molecular function, and cellular component. The left y-axis indicates the percentage of a specific GO category in that main category. The right y-axis indicates the annotated gene number expressed in a given sub-category. The downregulated DEGs are represented by blue, and the upregulated DEGs are represented by red.

For a broader classification, GO terms were assigned to classify all of the detected genes into different functional categories. A total of 56,198 out of the 62,562 detected genes were assigned to at least one GO annotation (Table S5). Furthermore, all of the assigned GO terms were categorized into 37 secondary functional groups under three main GO classification categories, including 16 functional groups for biological process, 9 for cellular component, and 12 for molecular function (Figure 2A). The biological process category suggested that large numbers of genes related to “metabolic process,” “cellular process,” “single-organism process,” “localization,” “response to stimulus,” “biological regulation,” and “signaling” were expressed during seedling PR development (Figure 2A). In the cellular component category, the terms “cell” and “cell part” were highly represented (Figure 2A). In the molecular function category, a significant number of genes were grouped into the “binding” and “catalytic activity” categories (Figure 2A; Table S5).

**Figure 3:**
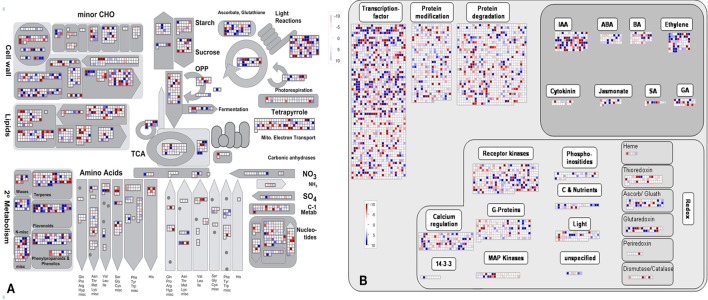
**The MapMan overview of cellular metabolism (A) and regulation (B) showing all of the detected genes in ***B. napus*** PR**. Individual genes are represented by small squares. The scale from −10 to +10 represents the average FPKM normalized log2 transformed counts of a detected gene in the four libraries.

In addition, the KEGG database was used to identify potential biological pathways represented in the *B. napus* PR transcriptome. A total of 12,424 detected genes assigned to 270 KEGG pathways (Table S6). KEGG analysis indicated that a greater number of genes expressed in *B. napus* PR were associated with metabolism, biosynthesis, plant hormone signal transduction, and several signaling pathways (Table S6). Furthermore, to obtain an overview of the metabolic and regulatory pathways in which the detected genes function, the 62,562 genes were analyzed using the MapMan software based on classification into 35 major pathways and 237 branch pathways. In cellular metabolism visualization, most of the genes were involved in cell wall metabolism, lipid metabolism, carbohydrate metabolism, and secondary metabolism (Figure 3A). In regulation visualization, a greater number of genes were categorized as protein modification- and degradation-related genes, plant hormone pathway-related genes, and signaling pathway-related genes (Figure 3B). Taken together, these functional categories indicated that high metabolic activities, regulation and signal transduction were required for cell developmental processes during PR growth.

### Differentially expressed genes (DEGs) between long and short PRL groups

The correlations among the four samples were calculated by PCA analysis and sample-to-sample cluster analysis based on the gene expression levels. The results suggested that the correlation between two individuals within the same groups was higher than that between individuals from different groups (Figure S3), indicating sufficient accuracy for the two biological replicates used in the present experimental design.

**Figure 4:**
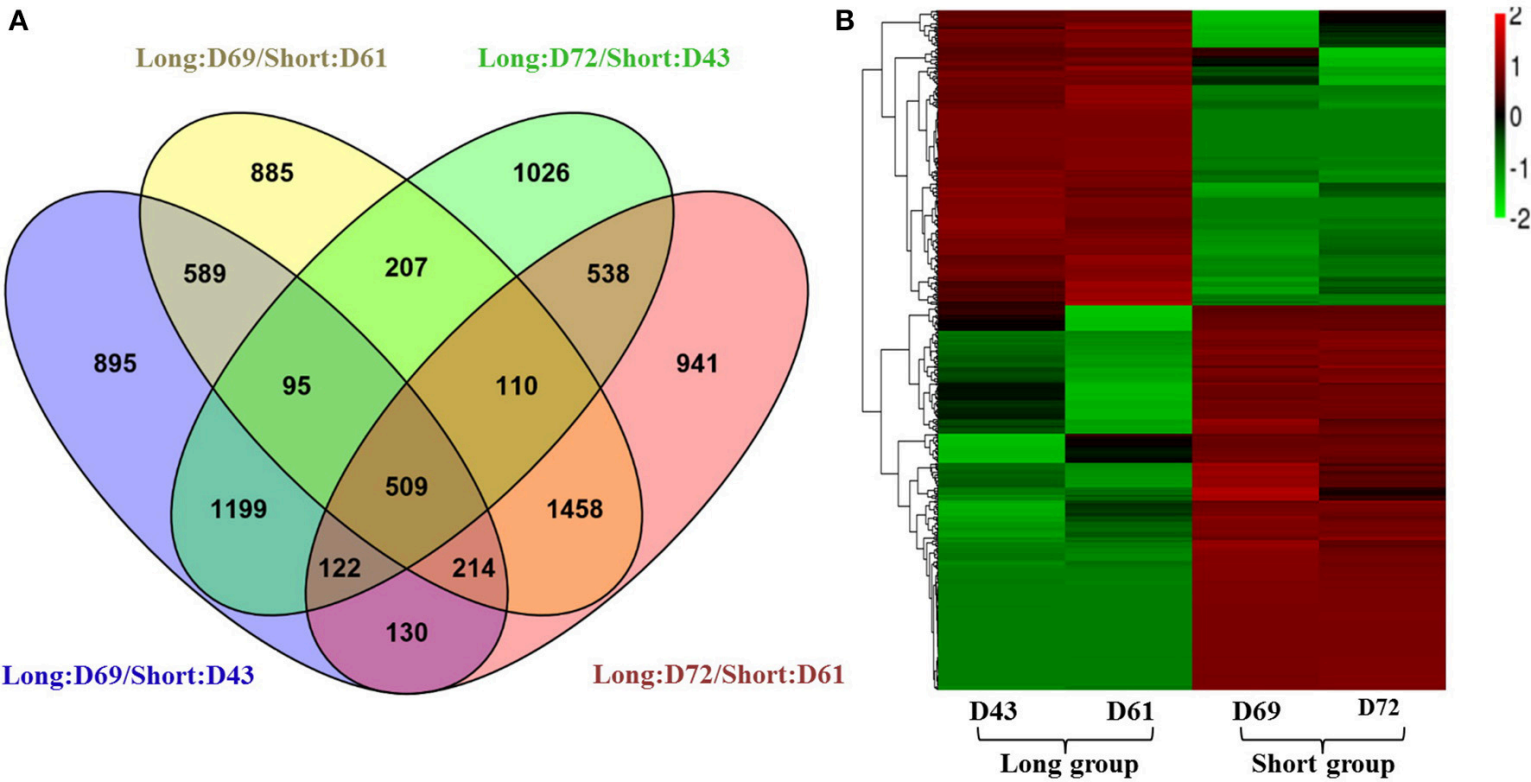
**Expression analyses of differentially expressed genes (DEGs). (A)** A Venn diagram showing the DEGs between two individuals from different PR groups (D69 and D43, D69 and D61, D72 and D43, and D72 and D61), and the overlapping DEGs among the four comparisons. **(B)** Hierarchical cluster analysis of 509 DEGs using normalized FPKM values. Red represents upregulated genes, and green indicates downregulated genes. For the normalization, the FPKM value of a DEG in each library first minuses the average FPKM value of the DEG in the four libraries; then divided by the FPKM standard deviation f the DEG in the four libraries. The final normalized FPKM values were range from −2 to +2.

To identify gene responses to PRL differences in *B. napus*, we used the DESeq method to examine the DEGs between the long and short groups using a pairwise approach, which can eliminate the background noise of the genotype-specific transcriptome to obtain more relevant data from the two groups. First, DEGs between two individuals from the long and short groups were identified with a threshold of absolute value of log_2_ fold-change ≥1 and *p* ≤ 0.05. Under these criteria, 3753, 4067, 3808, and 4023 genes exhibited significant differential expression between D69 and D43, D69 and D61, D72 and D43, and D72 and D61, respectively (Figure 4A). Then, 509 common DEGs from the four datasets were identified using a Venn diagram (Figure 4A). Details of the DEGs, their full names, log_2_ fold-change, and *p*-values in each pairwise comparison, as well as homologs and corresponding annotated information in *A. thaliana*, are presented in Table S7. Compared to the libraries from the long group, 288 genes were downregulated and 221 genes were upregulated in the libraries of the short group. These genes belong to diverse functional groups, including glycosyl hydrolase, kinases, phosphatases, cytochrome P450 family proteins, oxygenases, and hormone-responsive proteins, among others. A heat map was constructed to cluster the 509 DEGs based on the similarity and diversity of expression profiles using normalized FPKM values within a range of −2 to 2 (Figure 4B). The heat map clearly exhibited two clusters for the gene expression pattern of DEGs; the above cluster displayed DEGs upregulated in the short group, and the below cluster displayed DEGs downregulated in the short group (Figure 4B).

### Validation of DEGs using quantitative real-time polymerase chain reaction (qRT-PCR)

**Figure 5:**
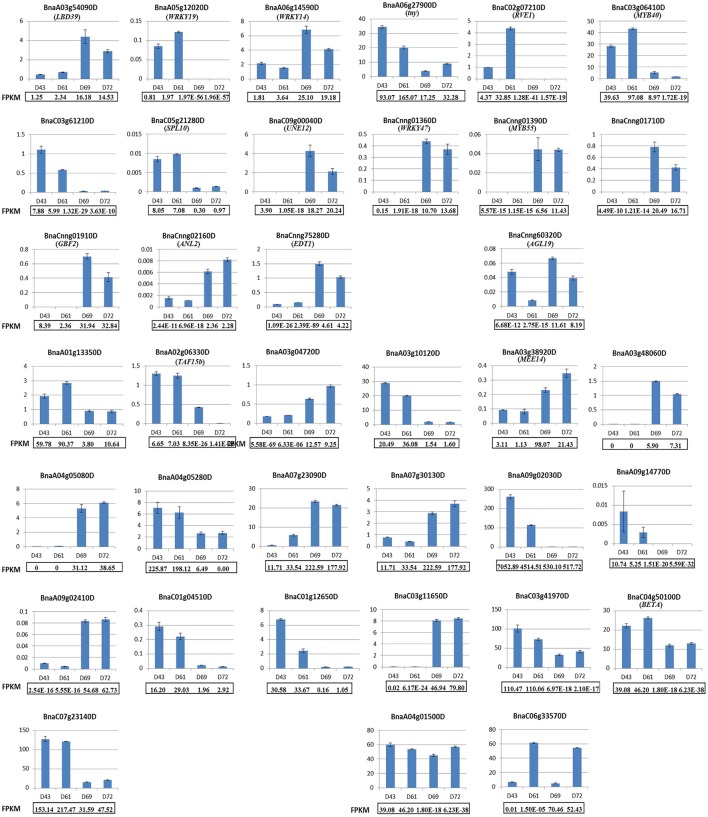
**Validation of 16 differentially expressed putative TFs and 21 randomly chosen DEGs by qRT-PCR**. The top three rows display the qRT-PCR results for 16 differentially expressed putative TFs. The last four rows display the qRT-PCR results for 21 randomly chosen DEGs. All of the genes except for *BnaCnng60320D, BnaA04g01500D*, and *BnaC06g33570D* exhibited consistently expressed patterns between qRT-PCR and RNA-seq analyses. The relative mRNA levels of the three biological replicates were calculated using the 2^−ΔΔCT^ method. The gene name for the selected DEG was shown in the parenthesis if it has been annotated by BLASTN in *Arabidopsis*.

To assess the validity and reliability of our RNA-seq data in identifying PRL-related DEGs, qRT-PCR was performed using gene-specific primers for all 16 of the differentially expressed putative TFs between two PR groups (for detailed information on the differential expressed TFs, see **Table 3** below) and 21 randomly chosen DEGs, which included genes encoding proteins with homologs that were previously functionally or non-functionally annotated in *Arabidopsis* and novel genes in *B. napus* (Table S2). Among these DEGs, the qRT-PCR profiles of 15 putative TFs and 19 randomly chosen genes mostly agreed with those obtained from the original RNA-seq results, although the expression fold-change displayed minimal differences, and only a putative TF and two randomly chosen DEGs differed in one or two samples (Figure 5). The expression patterns of these selected DEGs in the four genotypes between RNA-seq and qRT-PCR analyses displayed high similarity, thereby confirming the reliability of our RNA-seq data.

### Functional characterization of DEGs

**Figure 6:**
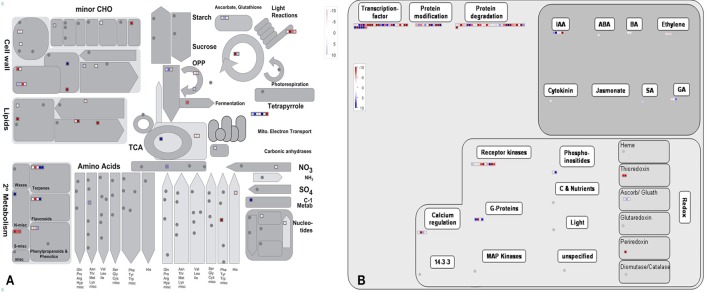
**The MapMan overview of cellular metabolism (A) and regulation (B) showing the DEGs those are responsible for PRL differences**. Individual genes are represented by small squares. The color key represents the average counts of log2 fold-change between the short and long PR groups. Red represents upregulation and blue represents downregulation between two PR groups.

GO classification was examined to gain functional information on the DEGs. In total, 148 upregulated DEGs and 173 downregulated DEGs were assigned to GO terms (Table S8). These GO classifications were categorized into 32 and 27 level-2 GO terms, respectively, under three top-level ontologies (Figure 2B). Consistent with GO classification with all of the detected genes, the top three GO terms of biological process for both upregulated and downregulated DEGs were “metabolic process,” “cellular process,” and “single-organism process” (Figure 2B). In the molecular function category, the predominantly overrepresented GO terms for upregulated and downregulated DEGs were “binding” and “catalytic activity.” In the cellular components category, “cell part,” “cell,” “membrane,” and “organelle” were highly represented for both upregulated and downregulated DEGs (Figure 6). Further GO subcategory analysis of DEGs provided clues to understand the detailed function of DEGs. The results revealed that “oxidation-reduction process,” “protein phosphorylation,” “carbohydrate metabolic process,” “signal transduction,” and “transport” were significantly enriched with both downregulated and upregulated DEGs (Table 2 and Table S8). Using REVIGO analysis, these GO terms were visualized by removing redundant GO terms. Highly similar GO terms and relevant GO terms are linked by lines in the graph of Figure S4, where the line width indicates the degree of similarity. The representative terms for downregulated DEGs were “phosphatidylcholine biosynthesis,” “ATP synthesis coupled proton transport,” “transport” “translation” “signal transduction” and “cell wall modification” (Figure S4B). For the upregulated DEGs, the representative terms were “protein phosphorylation” “translation” “response to oxidative stress” and “metal ion transport” (Figure S4B). These analyses demonstrated that DEGs encoding diverse metabolic, regulatory, signaling and structural proteins related to metabolic activity and cell expansion and/or proliferation were responsible for the PRL differences between the short and long groups.

**Table 2:** **The detailed GO subcategory analysis and KEGG pathways for DEGs in the RNA-seq analysis**.

**Top 10 of detailed GO subcategory analysis**		**Top 10 of KEGG pathways**	
**Downregulated DEGs**	**Upregulated DEGs**	**Downregulated DEGs**	**Upregulated DEGs**
Oxidation-reduction process (22)	Oxidation-reduction process (24)	Carbon metabolism (7)	Phenylpropanoid biosynthesis (6)
Protein phosphorylation (14)	Transmembrane transport (9)	Ribosome (6)	Carbon metabolism (5)
Proteolysis (11)	Carbohydrate metabolic process (8)	Pyruvate metabolism (5)	Calcium signaling pathway (4)
Carbohydrate metabolic process (8)	Response to oxidative stress (6)	Protein processing in endoplasmic reticulum (3)	Thyroid hormone signaling pathway (4)
Metabolic process (5)	Lipid metabolic process (6)	N-Glycan biosynthesis (3)	Phosphatidylinositol signaling system (4)
Translation (5)	Signal transduction (6)	Glucagon signaling pathway (3)	Oxidative phosphorylation (4)
Biosynthetic process (4)	Transport (6)	Carbon fixation in photosynthetic organisms (3)	Protein processing in endoplasmic reticulum (3)
Signal transduction (4)	Metabolic process (6)	Fatty acid metabolism (3)	Carbon fixation in photosynthetic organisms (3)
Transport (4)	Protein phosphorylation (6)	Two-component system (2)	Neurotrophin signaling pathway (3)
Intracellular protein transport (3)	Defense response (5)	Histidine metabolism (2)	Cysteine and methionine metabolism (3)

By comparing with the whole transcriptome background, 58 downregulated and 55 upregulated DEGs were assigned to KEGG pathways. Among these pathways, “carbon metabolism,” “ribosome,” “pyruvate metabolism,” “protein processing in endoplasmic reticulum,” and “N-Glycan biosynthesis” were the top five pathways represented by the downregulated DEGs (Table 2 and Table S9). In contrast, the majority of upregulated DEGs mapped to “phenylpropanoid biosynthesis,” “carbon metabolism,” “calcium signaling pathway,” “thyroid hormone signaling pathway,” “phosphatidylinositol signaling system,” and “oxidative phosphorylation” (Table 2 and Table S9). Furthermore, an overview of the DEGs between the short and long PR groups was visualized within a metabolic map and a regulated map using the MapMan software. In the cellular metabolism visualization, pathways showing significant changes in gene expression between the short and long PR groups included cell wall metabolism, carbohydrate metabolism, lipid metabolism, and secondary metabolism. In the regulation visualization, a greater number of DEGs were mapped to protein modification and degradation, hormone pathways (including auxin, abscisic acid, brassinosteroids, ethylene, salicylic acid, and gibberellin) and cell signaling (including receptor kinases, calcium, phosphoinositides, and G-proteins; Figure 6). A more detailed list of all of the DEGs corresponding to MapMan functional categories is displayed in Table S10. The MapMan and KEGG analyses were comparable to each other and validated the GO enrichment analysis. Combining MapMan and KEGG pathway analysis results with GO functional categories indicated that the DEGs that were related to cell wall metabolism, carbohydrate metabolism, lipid metabolism, secondary metabolism, protein modification and degradation, hormone pathways and signaling pathways were the main causes of the observed PRL differences between the short and long groups.

### Differentially expressed TFs associated with morphologic changes in the *B. napus* PR

The transition of cell fate in the RAM that determines the length and direction of PR requires the regulation of specific TFs (Giehl et al., 2014). We identified 16 differentially expressed TFs, including six upregulated TFs and 10 downregulated TFs, between the short and long PR libraries (Table 3). qRT-PCR analyses also demonstrated that the expression levels of theses TFs could be potentially related to morphologic changes in the *B. napus* PR. In addition, the homologs in *Arabidopsis* of several differentially expressed TFs are associated with PR development, such as the two putative TFs *REVEILLE 1* (*RVE1*) and *TINY* (Sun et al., 2008; Rawat et al., 2009). The overexpression of *TINY* and *RVE1* inhibits PR growth in *Arabidopsis*. Interestingly, the expression of *RVE1* (*BnaC02g07210D*) and *TINY* (*BnaA06g27900D*) was extremely enhanced in the short PR group. The differentially expressed TFs identified in this study are good candidates for further investigations about their potential regulating roles in *B. napus* PR development.

**Table 3:** **Differentially expressed transcript factors (TFs) in the RNA-seq analysis**.

**GeneID**	**Homologs in Ath**	**TF family**	**Short description**	**D43/D69**		**D61/D69**		**D43/D72**		**D61/D72**		**Regulated**
				**log_2_ fold change**	***p*-value**	**log_2_ fold change**	***p*-value**	**log_2_ fold change**	***p*-value**	**log_2_ fold change**	***p*-value**	
BnaA03g54090D	AT4G37540.1	LBD39	LOB domain-containing protein 39	−3.72	0.002	−2.77	0.039	−3.59	0.003	−2.63	0.036	Down
BnaA06g14590D	AT1G20700.1	WOX14	WUSCHEL related homeobox 14	−3.80	5E-04	−2.74	0.026	−3.43	0.002	−2.37	0.044	Down
BnaC09g00040D	AT4G02590.1	UNE12	Basic helix-loop-helix (bHLH) DNA-binding superfamily protein	−2.22	0.019	-Inf	1E-06	−2.39	0.008	-Inf	4E-08	Down
BnaCnng01360D	AT4G01720.1	WRKY47	WRKY family transcription factor	−6.02	3E-06	-Inf	1E-06	−6.40	7E-08	-Inf	2E-08	Down
BnaCnng01390D	AT4G01680.2	MYB55	myb domain protein 55	-Inf	2E-04	-Inf	7E-04	-Inf	5E-07	-Inf	4E-06	Down
BnaCnng01710D	AT4G00940.1		Dof-type zinc finger DNA-binding family protein	-Inf	1E-08	-Inf	7E-07	-Inf	6E-08	-Inf	7E-07	Down
BnaCnng01910D	AT4G01120.1	GBF2	G-box binding factor 2	−1.92	0.014	−3.73	7E-04	−1.99	0.008	−3.79	1E-04	Down
BnaCnng02160D	AT4G00730.1	AHDP	Homeobox-leucine zipper family protein	-Inf	5E-04	-Inf	0.002	-Inf	4E-04	-Inf	9E-04	Down
BnaCnng60320D	AT4G22950.1	AGL19	AGAMOUS-like 19	-Inf	0.008	-Inf	0.015	-Inf	0.021	-Inf	0.028	Down
BnaCnng75280D	AT1G73360.1	HDG11	Homeodomain GLABROUS 11	-Inf	0.013	-Inf	0.022	-Inf	0.012	-Inf	0.018	Down
BnaA05g12020D	AT4G12020.2	WRKY19	Protein kinase family protein	Inf	0.018	Inf	9E-04	Inf	0.01	Inf	2E-04	Up
BnaA06g27900D	AT5G25810.1	tny	Integrase-type DNA-binding superfamily protein	2.44	0.001	3.30	9E-04	1.51	0.024	2.38	0.004	Up
BnaC02g07210D	AT5G17300.1	RVE1	Homeodomain-like superfamily protein	Inf	0.002	Inf	2E-09	Inf	7E-04	Inf	3E-11	Up
BnaC03g06410D	AT5G14340.1	MYB40	myb domain protein 40	2.14	0.01	3.47	6E-04	Inf	3E-12	Inf	2E-14	Up
BnaC03g61210D	AT4G37850.1		Basic helix-loop-helix (bHLH) DNA-binding superfamily protein	Inf	6E-05	Inf	0.002	Inf	2E-05	Inf	5E-04	Up
BnaC05g21280D	AT1G27370.1	SPL10	Squamosa promoter binding protein-like 10	4.63	0.001	4.49	0.01	3.06	0.012	2.92	0.037	Up

WOX TF genes mainly function in the establishment of cell fate during embryogenesis (Haecker et al., 2004). *AtWOX14*, which acts downstream of the *PHLOEM INTERCALATED WITH XYLEM* (*PXY*) receptor kinase to promote vascular cell division, affects PR elongation (Etchells et al., 2013). *BnaA06g14590D*, homologous to *WOX14*, is downregulated in short PR libraries. MYB proteins that are present in all eukaryotes act as key factors in regulatory networks that control cell fate and identity (Dubos et al., 2010). Two DEGs, *BnaCnng01390D* (encoding MYB55), and *BnaC03g06410D* (encoding MYB40), belonging to the MYB family were expressed in PRs. The bHLH TFs maintain the balance between cell proliferation and differentiation in the *Arabidopsis* RAM and subsequently regulate the root growth rate (Makkena and Lamb, 2013). Our RNA-seq data discovered two bHLH DEGs, *BnaC09g00040D*, and *BnaC03g61210D*, homologous to *unfertilized embryo sac 12* (*UNE12*) and *AT4G37850*, respectively. Several TFs that regulate root growth are targeted by miRNAs. For instance, Class III HOMEODOMAIN-LEUCINE ZIPPER (HD-ZIP III) TFs restricted by the endodermis-specific expression of miR166b/165a are necessary for radial patterning and the proper growth of PRs in *Arabidopsis* (Carlsbecker et al., 2010; Miyashima et al., 2011). Two downregulated DEGs, *BnaCnng02160D* and *BnaCnng75280D*, which were both annotated as HD-ZIP, are hypothesized to be involved in the regulation of root growth rate. In summary, these differentially expressed TFs involved in cell fate decisions, including cell division, specification expansion, and elongation, may be important regulated factors that are related to morphological changes in the *B. napu*s PR.

### DEGs involved in carbohydrate, cell wall, and lipid metabolism

Roots rapidly grow to significantly alter root architecture and to capture more nutrients and water. This is evident by the changes in the expression of genes involved in carbohydrate metabolism, cell wall and lipid metabolism (Rasheed et al., 2016). To further determine whether the molecular and biochemical levels of carbohydrate, cell wall and lipid metabolism were significantly altered between the two PR groups, we analyzed detailed changes in the expression of related genes (Table S11). Pathway analysis revealed differences in the expression of genes involved in carbohydrate metabolism, including citrate cycle (TCA cycle), glycolysis, galactinol and galactose metabolism. The expression of five TCA cycle-related genes, three glycolysis metabolism-related genes, one galactinol metabolism-related gene and one galactose metabolism-related gene was altered (Table S11), which suggests that energy and carbon utilization blocks are required for PR growth at the seedling stage. Of five TCA cycle-related genes, two (*BnaA03g03440D* and *BnaCnng02240D*) were downregulated and three (*BnaA09g51860D, BnaC05g36200D*, and *BnaC07g13050D*) were upregulated (Table 2). NADP-ME2 (*BnaA03g03440D*) is the only cytosolic isoform, ubiquitously expressed NADP-dependent malic enzyme and is responsible for reversible malate decarboxylation, which affects root development in *Arabidopsis* (Badia et al., 2015). Of three glycolysis metabolism-related genes, *BnaA03g36910D* (*PKP1*) was downregulated, and *BnaC01g40210D* (*GAPC1*) and *BnaC02g44120D* were upregulated. *GAPC1* is a ubiquitous NAD-dependent glyceraldehyde-3-phosphate dehydrogenase, and the activity of this gene is restricted to elongating and differentiating cells (Rius et al., 2006; Henry et al., 2015). Moreover, the expression of *BnaC09g00800D* (*GSL5*, glucan synthase-like 5) and *BnaCnng62740D* (*AGAL1*, alpha-galactosidase 1) was significantly downregulated. *AGAL1* has been detected in quiescent center (QC) cells from the root meristem (Nawy et al., 2005).

Additions and re-arrangements of cell wall components are particularly required throughout growth and development, such as for cell elongation and cell proliferation in developing roots (Irshad et al., 2008). The primary cell wall, which is mainly composed of xyloglucans, is generally synthesized during cell expansion at the beginning of development (Goujon et al., 2003). Xyloglucan endotransglucosylase/hydrolase (XTH) proteins function in coordinating with cell expansion and elongation as key limiting enzymes for the modification of cell wall structure by cleaving and re-ligating xyloglucan polymers (Vissenberg et al., 2005; Shin et al., 2006). Three DEGs, *BnaC09g00910D* (*XTH9*), *BnaA10g29070D* (*XTH12*), and *BnaA02g35250D* (*XTH22*), control the xyloglucan metabolic pathways. Beta-xylosidase activity has been reported to be rate-limiting in xylan hydrolysis, which is involved in the secondary thickening of the cell wall (Goujon et al., 2003). *BXLs* (*BXL1, 2*, and *4*) encoding beta-xylosidase are involved in secondary cell wall metabolism (Goujon et al., 2003). The expression of *BnaA10g01280D* (*BXL2*) was downregulated in the short PR group. In addition to XTHs and BXLs, glycosyl hydrolase have been shown to be involved in the metabolism of xyloglucans and xylan, thereby contributing to cell wall extensibility (Shin et al., 2006). We also identified *BnaA06g17690D*, which encodes a glycosyl hydrolase family protein related to cell wall degradation and is upregulated in the short PR group. The expression of the cellulose synthase gene *BnaC03g03990D* (*CESA5*) was significantly upregulated in the short group. *CESA5* has been reported to contribute to secondary cell wall synthesis (Mendu et al., 2011). Moreover, *BnaA03g33850D* (*EXP12*, expansin 12) belonging to the alpha-expansin gene family was upregulated. The expansins are known to have cell wall loosening activity that is involved in cell growth and cell wall disassembly (Lu et al., 2016). The expression of *BnaA09g13260D* (*LRX2*) was also upregulated in the short group. Leucine-rich repeat extensins (LRXs) contribute to cell wall modification and influence plant growth (Draeger et al., 2015).

Lipid metabolism is a highly coordinated process that involves FA synthesis, FA elongation, and lipid degradation (Troncoso-Ponce et al., 2011). The expression of six lipid metabolism-related genes differed between two PR groups; five of these genes were downregulated and only one was upregulated. *BnaA03g24880D* (*MEE54*), *BnaAnng41260D* (*SVL2*), and *BnaA03g37330D*, involved in lipid degradation, were downregulated. *BnaC06g22680D* (*FAB1*, fatty acid biosynthesis 1), a key ACP synthase II involved in fatty acid elongation, was downregulated. *FAB1* contributes to plant growth, as conditional mutants for this gene show an auxin-resistant phenotype (Hirano and Sato, 2011). The expression of *BnaC09g13540D* (*CDS1*, CDP-diacylglycerol synthase 1) was upregulated. CDP-diglycerol is the precursor of phosphatidylinositol (PI; Lin et al., 2004), which plays a critical role in regulating optimal root growth (Lou et al., 2007).

### DEGs involved in hormone metabolism and signaling pathways during morphologic changes in *B. napus* PR

In plants, positional signals are required for proper cell division and cell elongation (Delay et al., 2013). Phytohormones, ions, protein kinases, phosphatases, and a variety of target proteins are key molecular players of diverse signaling pathways that regulate root developmental processes by interplaying with specific TFs (De Smet et al., 2015; Plattner, 2015). Combined with a functional analysis, we investigated the expression profiles of DEGs involved in hormone and signaling pathways (Table S12) to improve our understanding of signaling during morphological changes in the *B. napus* PR.

Auxin directly regulates cell division, differentiation, and elongation during root growth (Petricka et al., 2012). *BnaA01g14030D*, encoding the auxin biosynthesis-related gene *Tryptophan Aminotransferase Related 2* (*TAR2*), showed lower expression levels in the short group. Primary auxin-induced gene families consist of Auxin/Indole-3-Acetic Acid (Aux/IAA), Gretchen Hagen3 (GH3), and SMALL AUXIN UP RNA (SAUR; Hagen and Guilfoyle, 2002). Aux/IAA proteins interact with TIR1/AFBs (TRANSPORT INHIBITOR RESPONSE 1/AUXIN SIGNALING F-BOX) to promote their ubiquitination degradation, which in turn activates the release of AUXIN RESPONSE FACTORS (ARFs; Tan et al., 2007). The DEG *BnaC09g00530D* encoding AFB1 was involved in the ubiquitination degradation of Aux/IAA proteins. Furthermore, AFB1 promotes the degradation of Aux/IAA proteins that are mediated by the CUL1 (cullin 1)-based core SCF (Skp, Cullin, F-box containing complex) ubiquitin ligase complexes (De Smet et al., 2015). Interestingly, *BnaA09g00890D* (encoding *CUL1*) was upregulated in the short group. The F-box protein Skp2 is an ubiquitylation target of the CUL1-based core SCF complexes (Wirbelauer et al., 2000). *BnaA04g08740D*, encoding the Skp2-like protein, may also mediate the ubiquitination degradation of Aux/IAA proteins. The expression of five auxin responsive genes was altered, including two downregulated DEGs, *BnaC03g18820D* and *BnaCnng05190D*, encoding *DRM2*; one downregulated O-fucosyltransferase family protein (*BnaC09g01100D*); and two upregulated SAUR-like auxin-responsive protein family members, *BnaA10g14720D BnaC02g21400D*. SAUR proteins are involved in cell proliferation and cell expansion through the modulation of auxin transport (Kong et al., 2013). The overexpression of *SAUR39* reduces root length in rice (Kant et al., 2009). Together with the gene expression patterns, *BnaA10g14720D* and *BnaA10g14720D* possibly regulate PR growth by reducing cell expansion/proliferation in the PR.

Apart from auxin, other phytohormones, such as cytokinins, abscisic acid (ABA), ethylene, gibberellins (Gas), and brassinosteroids (BRs), also play fundamental roles in root growth, mostly depending on their crosstalk with auxin or with each other (De Smet et al., 2015). We identified 11 DEGs related to brassinosteroid, ethylene, cytokinin, abscisic acid, gibberellin, and salicylic acid metabolism. Most of these genes were crucial for the biosynthesis of these hormones. Squalene monooxygenases (SQPs) are involved in the sterol and brassinosteroid biosynthetic pathway in plants (Chappell, 1995). Two upregulated genes, *BnaA02g32720D* and *BnaA04g06060D*, both encode SQP2, which is involved in the root epidermis differentiation pathway (Bruex et al., 2012). The expression of *BnaC06g03450D* (*DWF1*, the cell elongation protein) was downregulated in the short group. *DWF1* controls brassinosteroid biosynthesis and influences cell elongation in *Arabidopsis* (Du and Poovaiah, 2005; Vriet et al., 2013). 2-Oxoglutarate (2OG) and Fe(II)-dependent oxygenase (2OG oxygenase) are ubiquitous iron enzymes that are required for ethylene metabolism. The inhibitors of 2OG oxygenase retard plant growth (Rose et al., 2011). Three DEGs (*BnaA10g01910D, BnaA05g26300D*, and *BnaC03g73320D*), encoding 2OG oxygenase superfamily proteins, are related to ethylene metabolism. In addition, another ethylene biosynthesis-related gene, *BnaCnng67880D* (*ACO1, ACC oxidase 1*), was downregulated. 1-Aminocyclopropane-1-carboxylate (ACC) oxidases convert ACC to ethylene at the final step in ethylene biosynthesis (Hudgins et al., 2006). The expression of another four genes crucial for the metabolism of cytokinins, GAs, ABA, and salicylic acid were also significantly altered, including *BnaA10g24670D* (*UGT76C1, UDP-glucosyl transferase 76C1*), *BnaA07g38510D* (*GASA1, GAST1 protein homolog 1*), *BnaA09g55700D* (*MARD1*), and *BnaA03g31730D* (*BSMT1*). *UGT76C1* regulates cytokinin responses by cytokinin N-glucosylation in *Arabidopsis* (Wang et al., 2013). *GAST1* (*gibberellic acid stimulated transcript 1*) is a gibberellin-responsive factor that is possibly involved in cell elongation (Cui et al., 2016). *AtBSMT1* encodes a salicylic acid (SA) methyltransferase related to salicylic acid metabolism.

Cell signaling activates cellular signal transduction pathways to regulate cellular responses to the environment and other cells. We investigated the expression profiles of genes involved in several signaling pathways, including protein kinases, phosphatases, calcium, and phosphoinositides, which have been extensively reported to regulate root development. The expression of 19 protein kinases, 3 phosphatases, 4 calcium-related genes, and 3 phosphoinositide-related genes was altered between the long and short PR groups (Table S12). Phytohormones directly regulate receptor-like kinase signals by phosphorylating target proteins that are required for root development (De Smet et al., 2009). In the root, cytokinins induce cell mitosis by increasing the activity of D-cyclin-dependent kinase (CYCD-CDK) complexes that promote cell cycle progression (Randall et al., 2015). The downregulated DEG *BnaC09g00570D* encodes *CYCD6;1*, which is required for the asymmetric cell division of the cortex/endodermis initials in root (Sozzani et al., 2010). Furthermore, we identified the downregulated gene *BnaAnng30910D*, which encodes the cyclin-dependent protein kinase CDKB2;1. *CDKB2;1* is closely related to the maintenance of mitotic activity in developing roots in *Arabidopsis* (Okushima et al., 2014). Brassinosteroids interact with the leucine-rich-repeat receptor-like-kinase (LRR-RLK) *Brassinosteroid-Insensitive 1* (*BRI1*) and then phosphorylates BR-signaling kinases (BSKs) to activate the BR signal transduction cascade that mediates QC division (Kim and Wang, 2010). A component of the Brassinosteroids (BRs) signaling cascade, GSK3/shaggy-like kinase (GSK), controls cell elongation downstream of *BRI1* (Rozhon et al., 2010). The downregulated DEGs *BnaCnng02180D* (encoding BSK3) and *BnaCnng02170D* (encoding Shaggy-like protein kinase 32, SK32) are hypothesized to regulate BR signal transduction in the PR. The process of ABA-mediated PR growth is positively modulated by kinases but negatively controlled by phosphatases (Jiao et al., 2013). Protein phosphatase type 2C (PP2C) negatively regulates ABA-inhibited PR growth in *Arabidopsis* (Rubio et al., 2009). We identified three DEGs, *BnaA03g00600D, BnaA10g26970D*, and *BnaC09g00700D*, belonging to the PP2C family. Furthermore, the ABA signal transduction pathway also depends on the Ca^2+^ levels to suppress PR elongation in rice and *Arabidopsis* (Chen et al., 2006; Bai et al., 2007). Calmodulin is a conserved Ca^2+^ sensor protein and regulates the function of calmodulin-binding proteins, such as *DWF1* (Du and Poovaiah, 2005). The expression of *BnaA04g02760D* (*CAM3, calmodulin 3*) was upregulated. In addition, three DEGs *BnaA09g40510D* (*Iqd4, IQ-domain 4*), *BnaC04g11110D* (*CLO-3, caleosin 3*), and *BnaC01g13190D*, encoding calmodulin binding proteins, were identified in our data.

The phosphatidylinositol (PI) signaling system also plays a critical role in regulating optimal root growth (Lou et al., 2007; Rodriguez-Villalon et al., 2015). To start the PI signaling pathway, PI is synthesized from CDP-diglycerol and cytoplasmic inositol by PI synthases (PIS; Lin et al., 2004). *BnaC09g13540D* encodes CDP-diacylglycerol synthase 1 (CDS1), which is involved in PI biosynthesis. Subsequently, the sequential phosphorylation of PI by PI4-kinase (PI4K) and PI-phosphate 5-kinase (PIPK) forms the substrate of phospholipase C (PLC), which in turn activates the PI signal transduction cascade (Lin et al., 2004). The expression of *BnaC05g43070D* (encoding PIP5K9) was altered. The T-DNA insertion mutation of PI monophosphate 5-kinase (PIP5K9) results in shortened PRs due to reduced cell elongation in *Arabidopsis* (Lou et al., 2007). Two upregulated DEGs, *BnaUnng04440D* and *BnaA02g07530D*, were both homologous to *AtPLC4* and possibly affect phosphatidylinositol signal transduction during PR development. Collectively, these results suggest that signal transduction and hormone metabolism significantly affect PR growth in *B. napus*.

### A first insight into the genome-wide transcriptional landscape of the primary root in *B. napus*

The PR of plants plays a significant role in RSA formation and yield determination. Despite its importance, extensive genomic information on functional genes has not been pursued to improve the understanding of the molecular processes of PR development in rapeseed. In the present study, the application of RNA-Seq technology provides genome-wide transcriptional information for *B. napus* PR. It has been reported that in *B. rapa*, 32,335 genes (78.8% of the total 41,020 annotated genes) were expressed in at least one tissue and 18,876 expressed genes were constitutively expressed throughout the plant life cycles (Tong et al., 2013). In *B. napus*, an allopolyploid originating from *B. rapa* and *B. oleracea*, our data detected 62,562 genes (61.9% of the annotated genes), approximately twice the number of *B. rapa* genes that are expressed in the seedling PR. Thus, a majority of expressed genes in the *B. napus* PR are also likely required for all tissue development.

Global transcriptomic analyses help us to understand the molecular mechanism of the development of vital organs in crops, such as seeds in grain pea (Liu et al., 2015), embryos in *B. rapa* (Zhang et al., 2014), tuberous roots in *Rehmannia glutinosa* (Sun et al., 2015), and cluster roots in *Lupinus albus* (Wang et al., 2014). Gene expression profiles and functional annotation analyses demonstrated that the expression of genes associated with cellular process, metabolic processes, biological regulation, singling, and cellular component organization play critical roles in these developmental processes. Similar gene expression trends and biological pathways were observed in *B. napus* PR development, as suggested by GO, KEGG, and MapMan analyses. And the importance and generality of these pathways in root development have also demonstrated by proteomic analyses in rice (Wang et al., 2014) and *Arabidopsis* (Qian et al., 2015). Furthermore, at least 44,986 genes were actively expressed in each *B. napus* PRs in our data, suggesting conserved expression patterns for genes involved in PR development. Thus, our data provide an initial view of the PR developmental process, facilitating in the identification of candidate genes related to metabolism, hormone, and signaling pathways and many TF genes for a further understanding of the molecular mechanism of PR development.

### Overview of the changed biological processes responsible for PRL differences

**Figure 7:**
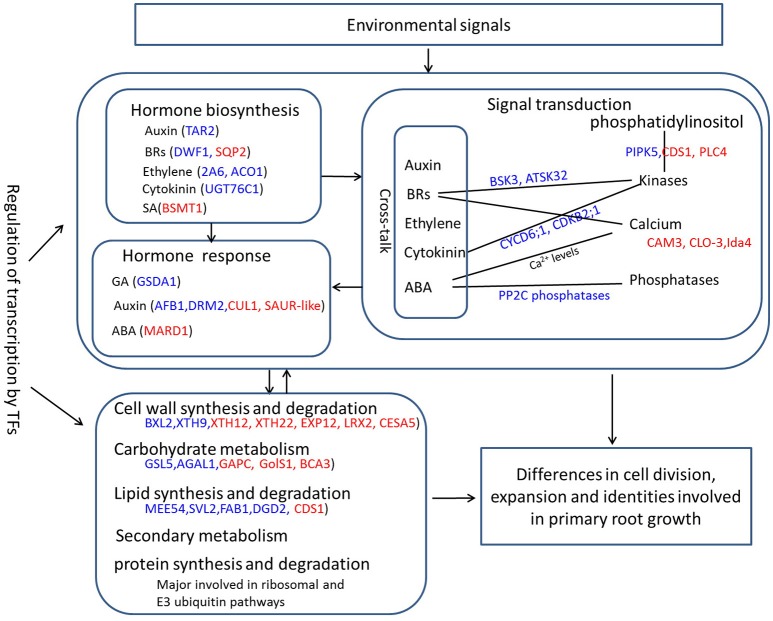
**A schematic diagram of overview of the changed biological processes and related genes responsible for PRL differences**. Red font represents upregulated genes, and blue front indicates downregulated genes.

In general, primary root growth starts with the division, differentiation and expansion of SCN cells residing in the RAM. The metabolic homeostasis of energy and substances is pivotal for cell developmental processes in all organisms. These metabolism products, usually carbohydrates, proteins, and lipids, are able to stabilize cellular structures that are required for root growth (Braybrook and Harada, 2008; Park and Harada, 2008). In the present study, the carbohydrate, protein, lipid and cell wall metabolism pathways have been significantly changed between two PR groups, as suggested by functional analyses of DEGs. However, primary root development is a highly complex process that is coordinated by hormone synthesis, signal transduction, metabolism and their crosstalk (De Smet et al., 2015). Based on the analyses in this study, we proposed a putative model for the overview of the changed biological processes responsible for PRL differences, which are in part schematically displayed in Figure 7. By detecting environmental signals, the PR developmental process initiates under the intervention of several crosstalks, which involve hormone synthesis, signal transduction, metabolism, and regulation of a series of PR development-related genes that has been widely reported (Petricka et al., 2012). In the past few decades, the biosynthesis and transport of major plant hormones (auxin, ABA, BAs, GA, cytokinin, and ethylene) have been found to contribute significantly to primary root growth (De Smet et al., 2015). Numerous effects of hormones on stem cell maintenance, proliferation, and differentiation in PR development are the result of crosstalk between different hormonal pathways (De Smet et al., 2015). In the short PR group, the altered expression of several hormone biosynthesis- and response-related genes may inhibits the growth of the primary root. Protein degradation of hormone repressors and the activation of hormone responsive factors are common signaling pathways that are regulated by key molecular signals, such as kinases, Ca^2+^, cyclic nucleotides, protein kinases and phosphatases, and a variety of target proteins in interplay with specific TFs (Petricka et al., 2012). As described above, the expression levels of kinase-, Ca^2+^-, and phosphatase-related genes possibly involved in hormone signal transduction significantly changed. Then, the development signals trigger the regulation of the transcription of developmental genes and a series of biological processes including carbohydrate, cell wall, lipid, and protein metabolism. The supply of the metabolism products in the whole plant in turn induces hormone biosynthesis and signal transduction. Our study expands upon the current understanding of the molecular mechanism of primary root development in *B. napus*, and our findings will facilitate PR gene identification together with genome-wide association studies.

## Conclusions

In summary, a global comparative transcriptomic profile of two PRL group in *B. napus* was intensively investigated by transcriptome sequencing. The results provide valuable insight into the molecular basis of primary root development at global transcriptional levels. We showed that the PR development is a highly complex process that is regulated by several crosstalks including metabolism process, cellular process, response to stimulus, biological regulation, and signaling. Our large transcriptome dataset also provides information about the changed biological processes (hormone synthesis, signal transduction, and metabolism) and the key candidate genes responsible for primary root growth differences.

## Author contributions

XD performed the data analysis and wrote the main manuscript text. JW and ZT contributed to tissue collection, RNA extraction and quantitative RT-PCR. XW and GL prepared the plant materials. HW designed and managed the experiments and reviewed the manuscript. All of the authors have read and approved the final manuscript.

### Conflict of interest statement

The authors declare that the research was conducted in the absence of any commercial or financial relationships that could be construed as a potential conflict of interest.

## Supplementary material

Supplementary Material Presentation 1 **The Perl scripts used to clean the raw data and map the clean reads to the ***B. napus*** reference genome**.

Table S1 **Detailed information on 40 natural ***B. napus*** accessions, including their accession number, and genetic distances**.

Table S2 **Gene ID and gene-specific primers of 16 differentially expressed putative TFs and 21 randomly chosen DEGs for qRT-PCR validation**.

Table S3 **Information on the 62,562 genes detected in four cDNA libraries**.

Table S4 **Identified 5102 putative transcription factors (TFs) belonging to 59 TF families**.

Table S5 **Detailed subcategory GO enrichment analysis of all of the detected genes under three main GO classification categories**.

Table S6 **Detailed KEGG pathway enrichment analysis of all of the detected genes**.

Table S7 **Information on the 509 differentially expressed genes between the short group and the long group**.

Table S8 **Detailed subcategory GO enrichment analysis of 509 DEGs**.

Table S9 **Detailed KEGG pathway enrichment analysis of 509 DEGs**.

Table S10 **Detailed list of all of the DEGs corresponding to MapMan functional categories**.

Table S11 **Representative differentially expressed genes involved in carbohydrate metabolism, cell wall and lipid metabolism in the RNA-seq analysis**.

Table S12 **Representative differentially expressed genes involved in hormone pathways and signaling pathways in the RNA-seq analysis**.

Figure S1 **Distribution of gene coverage in the four libraries**.

Figure S2 **Venn diagram showing gene numbers expressed in the primary roots of four ***B. napus*** genotypes**.

Figure S3 **Relatively close PCA (A) and clustering distance (B) analysis revealed correlations among the four genotypes**. 43.2, D43; 61.2, D61; 69A.2, D69; and 72A.2, D72.

Figure S4 **The interactive graph view by REVIGO showing the relative GO terms enriched in downregulated DEGs (A) and upregulated DEGs (B)**. Bubble color indicates the *p*-value; bubble size indicates the frequency of the GO term in the underlying GOA database. Highly similar GO terms are linked by lines width indicates the degree of similarity.
