# Supplementary material for: Comparative Transcriptome Analysis of Primary Roots of Brassica napus Seedlings with Extremely Different Primary Root Lengths Using RNA Sequencing
Source: Front Plant Sci. 2016 Aug 19;7:1238. doi: 10.3389/fpls.2016.01238 (PMC4990598; doi:10.3389/fpls.2016.01238)
Supplement: Supplementary Material Presentation 1 — The Perl scripts used to clean the raw data and map the clean reads to the B. napus reference genome. [file Presentation1.pdf]

The self-written Perl script in NGS QC Toolkit v2.3.3 software used to clean the raw data

First, low-quality sequences (reads has more than 70% of low-quality bases with a quality value ≤ 20) were removing from the raw data:

perl $NGSQCToolkit/QC/IlluQC_PRLL.pl -pe R1.fastq R2.fastq N 5 -l 70 -s 20 -c 15

–o, rawdata_stat

-pe, Paired-end read files

N, Do not filter for Primer/Adaptor

5, Illumina (1.8+) (Phred+33, 33 to 74)

-c, Number of CPUs to be used

R1.fastq R2.fastq, input data

Second, the low-quality bases from the 3' terminus with a quality value ≤ 20 were removing:

perl $NGSQCToolkit/QC/IlluQC_PRLL.pl -pe R1.fastq R2.fastq N 5 -l 70 -s 20 -c 15

-o rawdata_stat

-i, Forward read/sequence file

-irev, Reverse read/sequence file of paired-end data

-q, qual CutOff

R1.fastq_filtered -irev R2.fq_filtered, input fastq data obtaining from the first step

Third, the uncertain bases (N) in all of the read with a length threshold ≤ 35bp were removing:

perl $NGSQCToolkit/Trimming/AmbiguityFiltering.pl -i R1.fastq_filtered_trimmed -irev R2.fastq_filtered_trimmed -t5 -n 35

-i, Forward read/sequence file

-irev, Reverse read/sequence file of paired-end data

-t5, Trim ambiguous bases from 5' end of the sequence

-n，Sequence length cut-off

R1.fastq_filtered_trimmed、R2.fastq_filtered_trimmed, input fastq data obtaining from the second step

Then, the clean data was obtained as the final output data from the third step.

The self-written Perl script used for the mapping of the clean reads to the *B. napus* reference genome

tophat -r 50 -o tophat --library-type fr-firststrand -p 15 genome R1.fq R2.fq

-r, --mate-inner-dist

--library-type, the type for the sequencing of the library

-p, Number of CPUs to be used

genome, name of the reference genome (The reference genome has been indexed first used the bowtie2-build)

R1.fq、R2.fq, input clean data
