# Supplementary figures and images for: Comparative Transcriptome Analysis of Primary Roots of Brassica napus Seedlings with Extremely Different Primary Root Lengths Using RNA Sequencing
Source: Front Plant Sci. 2016 Aug 19;7:1238. doi: 10.3389/fpls.2016.01238 (PMC4990598; doi:10.3389/fpls.2016.01238)

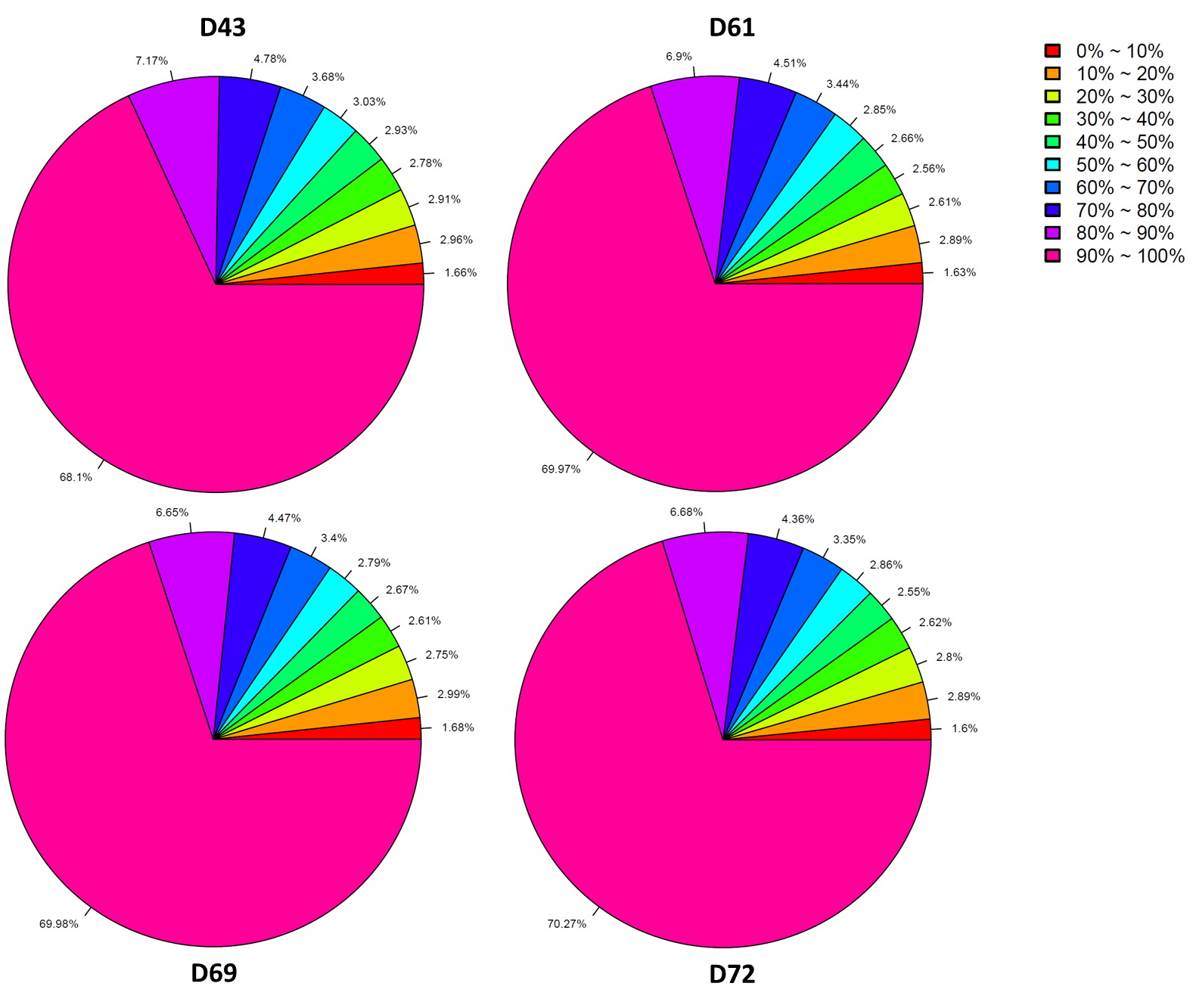

Supplement: Figure S1 — Distribution of gene coverage in the four libraries. [file Image1.JPEG]

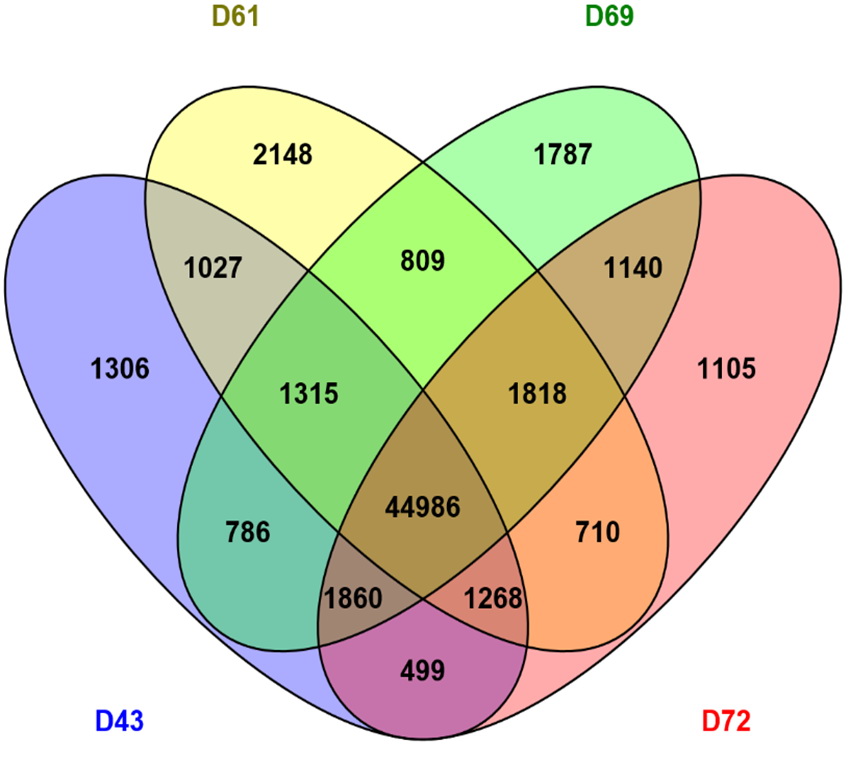

Supplement: Figure S2 — Venn diagram showing gene numbers expressed in the primary roots of four B. napus genotypes. [file Image2.JPEG]

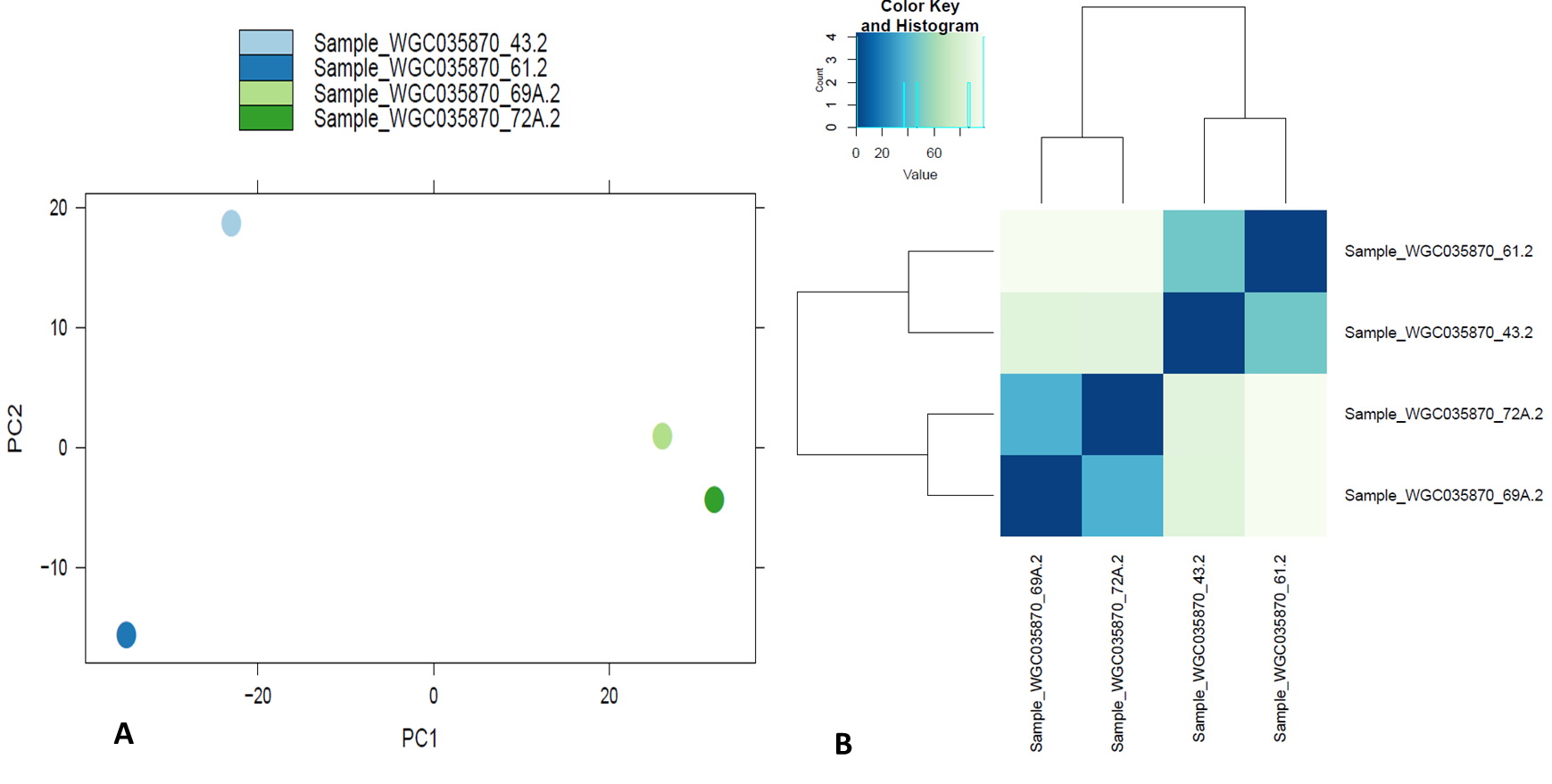

Supplement: Figure S3 — Relatively close PCA (A) and clustering distance (B) analysis revealed correlations among the four genotypes. 43.2, D43; 61.2, D61; 69A.2, D69; and 72A.2, D72. [file Image3.JPEG]

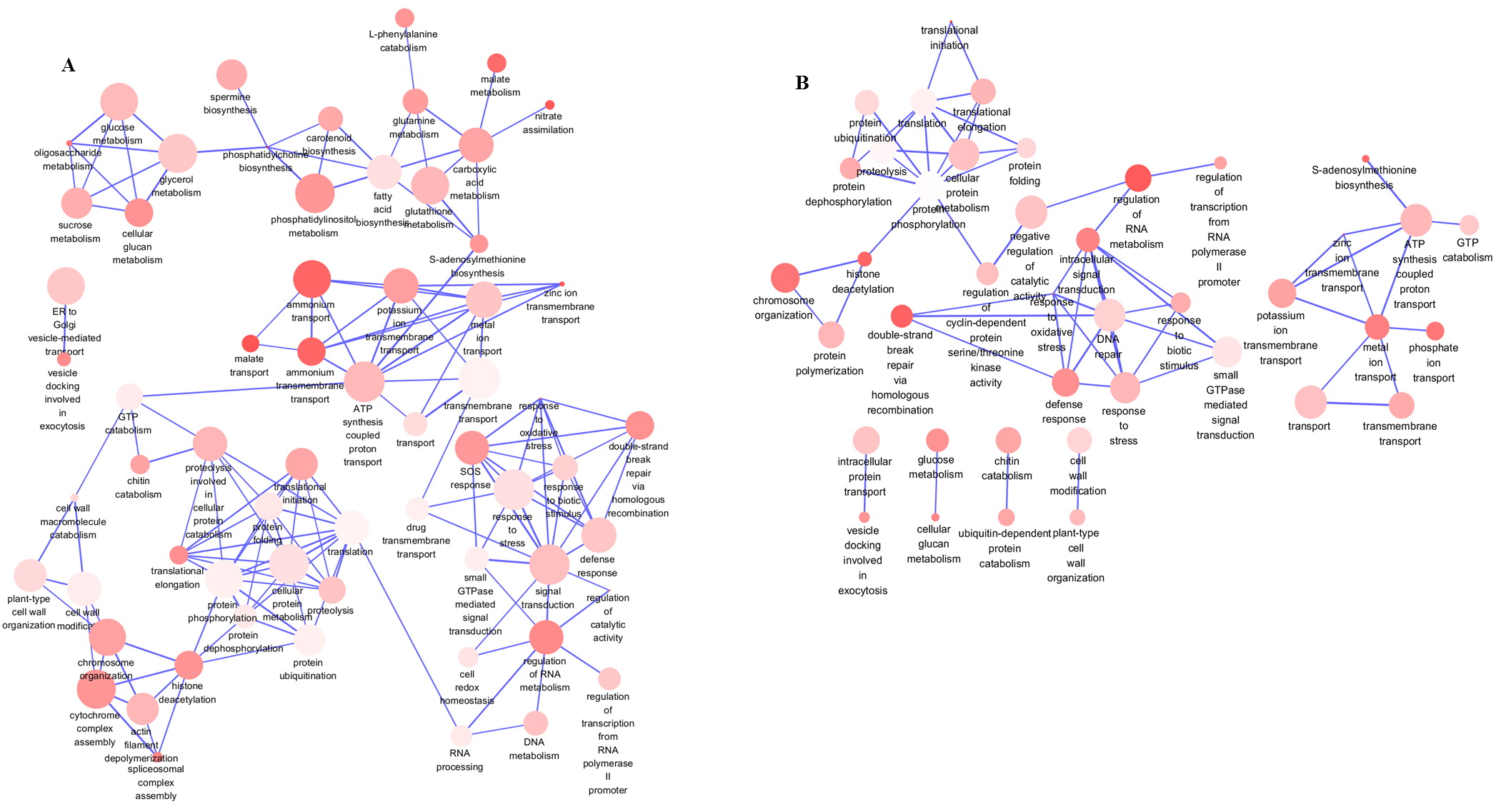

Supplement: Figure S4 — The interactive graph view by REVIGO showing the relative GO terms enriched in downregulated DEGs (A) and upregulated DEGs (B). Bubble color indicates the p-value; bubble size indicates the frequency of the GO term in the underlying GOA database. Highly similar GO terms are linked by lines width indicates the degree of similarity. [file Image4.JPEG]
